# GFRP Bars Anchorage Resistance in a GFRP-Reinforced Concrete Bridge Barrier

**DOI:** 10.3390/ma12152485

**Published:** 2019-08-05

**Authors:** Michael Rostami, Khaled Sennah, Saman Hedjazi

**Affiliations:** 1 Civil Engineering Department, Ryerson University, Toronto, ON M5B 2K3, Canada; 2 Civil Engineering and Construction Department, Georgia Southern University, 201 COBA Drive, Statesboro, GA 30460, USA

**Keywords:** GFRP bars, 180°-hook anchorage, concrete barrier, bridge barriers, barrier–deck junction, anchorage resistance, experimental testing

## Abstract

In the present paper, experimental and numerical investigations were conducted on concrete bridge barriers utilizing glass fiber reinforced polymer (GFRP) bars with a hook at their ends. Implementation of these hooked bars instead of the bent bars or headed-end bars in the bridge barriers presented in the Canadian Highway Bridge Design Code (CHBDC) was investigated on American Association for State Highway and Transportation Officials (AASHTO) test level 5 (TL-5) concrete bridge barriers. This research aimed to reach a cost effective and safe anchorage method for GFRP bars at the barrier–deck junction, compared to the conventional bend bars or headed-end bars. Therefore, an experimental program was developed and performed to qualify the use of the recently-developed, small radius hooked bars at the barrier–deck junction. The experimental findings were compared with the design factored applied transverse load specified in CHBDC for the design of the barrier–deck junction as well as factored applied bending moment obtained at the barrier–deck junction using a recently-conducted finite-element modeling. Satisfactory behavior for the developed hooked GFRP bars as well as their anchorage resistance was established and a reasonable factor of safety in design of barrier–deck joint was achieved.

## 1. Introduction

Bending of glass fiber reinforced polymer (GFRP) bars at construction sites is not possible, and bent bars do not have the same strength as straight bars. This problem results in an increase in the number of GFRP bars used in construction. Alternatively, headed-end GFRP bars can address this problem. A vehicle crash test was conducted using a AASHTO test level 5 (TL-5) bridge barrier reinforced with headed-end, sand-coated GFRP bars, and resulted in a new structural design for bridge barriers [1]. Another crash test was conducted on an actual size barrier reinforced with ribbed-surface GFRP bars, confirming the same design [2]. These crash tests were performed in accordance with the Manual for Assessing Safety Harware (MASH) test level 5, TL-5, [3], and showed acceptable resistance for the GFRP bars in sustaining vehicle impact [4]. Parts of the crash-tested barriers were tested further to collapse under transverse static loading. Their ultimate experimental load carrying capacities were observed to be far greater than the factored design loads specified in the AASHTO-LRFD specifications and the Canadian Highway Bridge Design Code [5,6]. Other studies were conducted to determine the transverse capacity of the steel-reinforced barrier using yield line analysis [7,8].

A design for precast ultra-high performance fiber-reinforced concrete (UHPFRC) TL-4 barriers, including investigation into their mechanical behavior under quasi-static transverse loading up to failure, was done both experimentally and using finite element modeling [9]. Experimental testing on cast-in-place and precast barriers subjected to quasi-static loading and anchored bridge decks was also done in 2015, with all tested configurations exceeding the design criteria in CSA-S6-06 and AASHTO LRFD specifications [10]. Finite element modeling was used in the comparison between the load transfer and failure mode of precast and cast-in-place bridge barriers [11]. Fiber-reinforced concrete has also been used in bridge barriers in other studies, revealing the mechanical properties of the barriers under static and dynamic loading [12]. Concrete railing and deck connection with internal FRP I-bars was investigated, incorporating static tests on the connection of the railing post and deck [13], and a design procedure for the concrete post to deck joint was proposed [14]. Anchorage capacity of precast concrete bridge barriers for test level 2 (TL-2) has been studied elsewhere, with finite element modeling used for the evaluation of capacity of TL-4 barriers [15].

**Figure 1:**
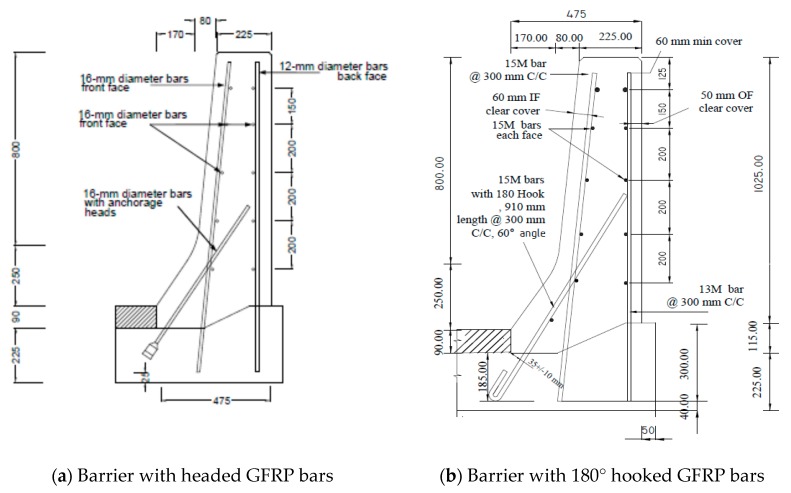
Reinforcement details of AASHTO test level 5 (TL-5) barrier reinforced with glass fiber reinforced polymer (GFRP) bars with headed ends and 180° hooks. (**a**) Barrier with headed GFRP bars; (**b**) Barrier with 180° hooked GFRP bars.

**Figure 2:**
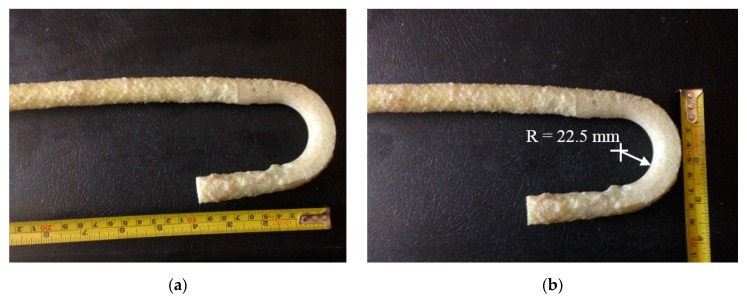
Views of the developed GFRP bar with 180° hook. (**a**) length of the hook; (**b**) radius of the hook.

Figure 1a shows the sand-coated GFRP bar details of the crash tested barrier utilizing headed-end bars at the barrier–deck junction [1]. The manufacturer of the headed-end bars has recently used a twisted roving method to develop a GFRP bar with a 180° hook with reduced radius, to reduce the cost associated with the use of the headed-end bars at the barrier–deck junction. An alternative design for the barrier wall is presented in Figure 1b, showing the revised details of the TL-5 barrier by replacing the headed bar shown in Figure 1a with a hooked bar. The developed hook shown in Figure 2 has an inner radius of 22.5 mm and a length of 100 mm. Table 1 summarizes the material properties of the GFRP bars used in the present study, as obtained from the manufacturer [16] The 185 mm vertical embedment length into the deck slab cantilever was considered given the fact that the typical slab thickness used by Ontario Ministry of Transportation in Ontario bridges is 225 mm, with the difference between then representing the proper concrete cover between the bottom of the bar and the bottom surface of the deck slab.

To qualify the revised barrier design in Figure 1b, experimental tests were conducted to ensure that the capacity of barrier–deck junction satisfied Canadian Highway Bridge Design Code (4) requirements. CHBDC specifies transverse, longitudinal, and vertical loads of 210, 70 and 90 kN, respectively, for design of a TL-5 barrier–deck anchorage. By inspection, the transverse load simulating vehicle impact is the only load out of these three load components that affects the barrier–deck joint design. As such, the factored design transverse load to be compared with the barrier experimental load carrying capacity was 357 kN, considering a 1.7 load factor, distributed over 2400 mm length in the direction of traffic and at a height of 990 mm over the deck slab top surface.

## 2. Experimental Study

**Figure 3:**
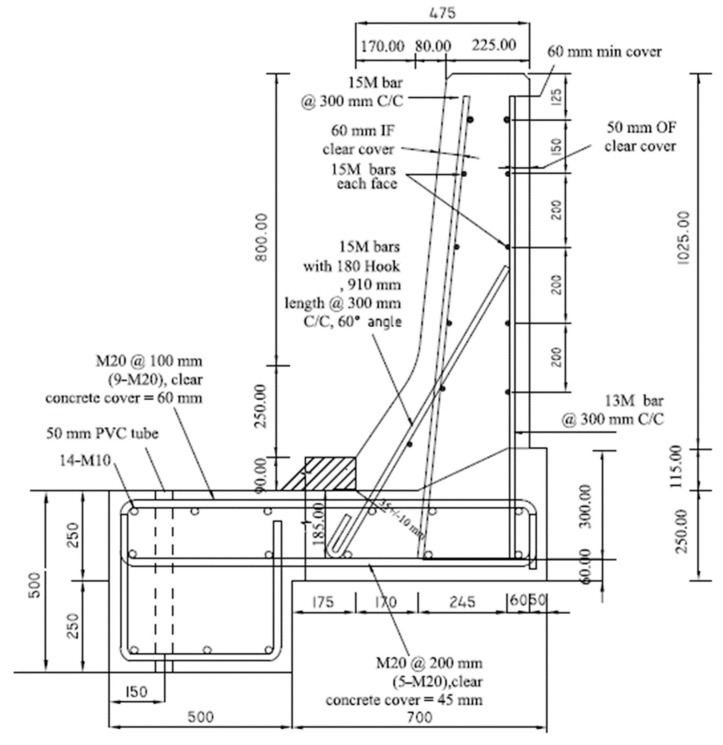
Specimen # 1: Interior location of barrier connected to a cantilever slab (hooks facing the roadway).

**Figure 4:**
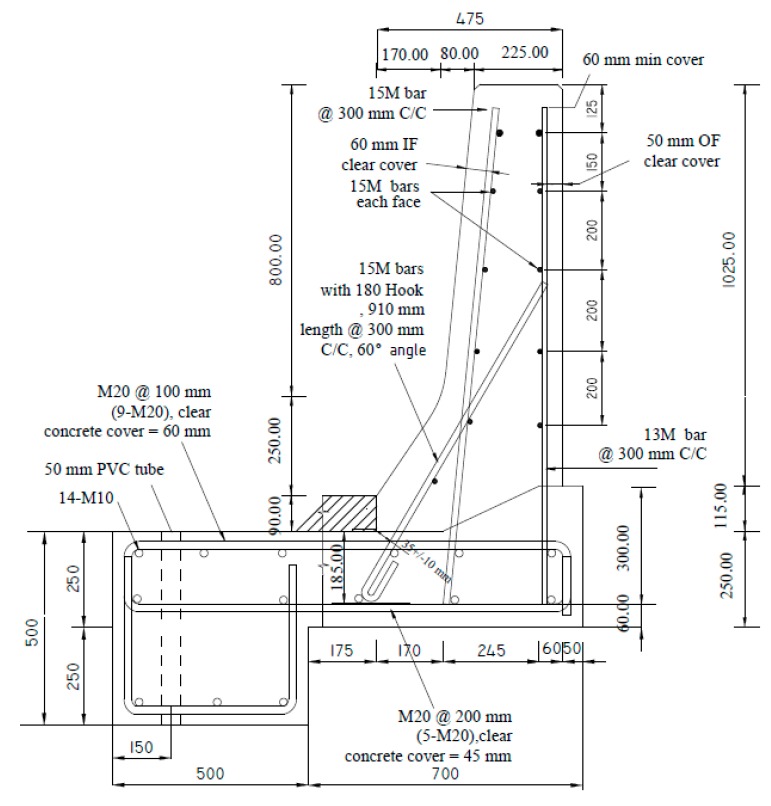
Specimen # 2: Interior location of barrier connected to a thin cantilever slab (hooks not facing roadway).

**Figure 5:**
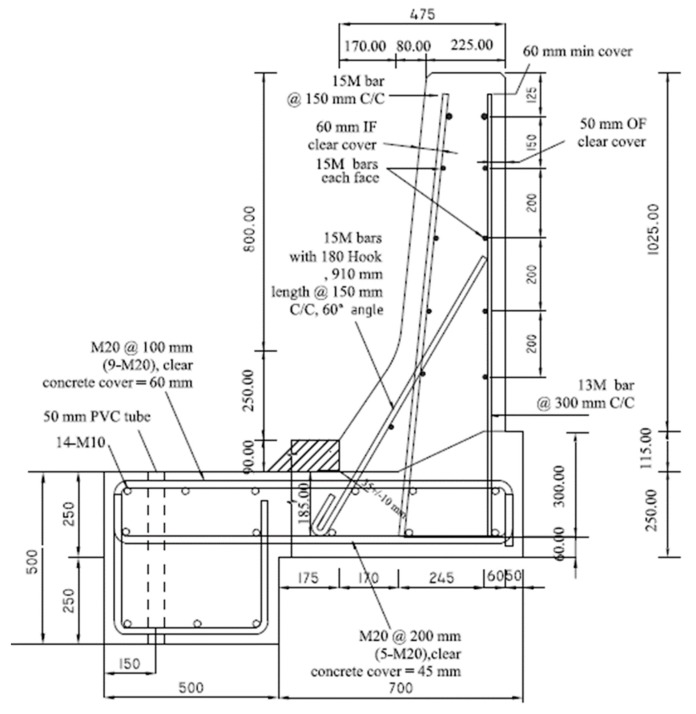
Specimen # 3: End location of barrier connected to a cantilever slab.

**Figure 6:**
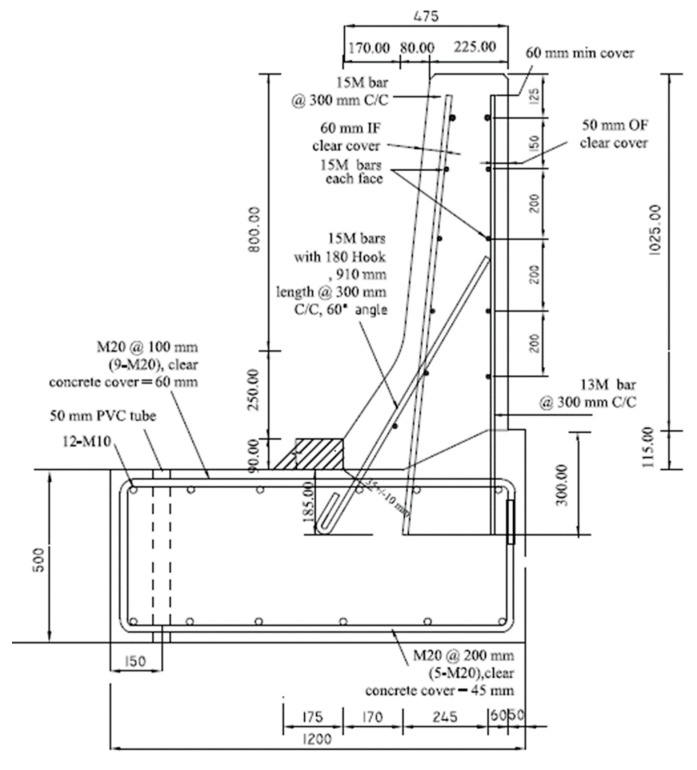
Specimen # 4: Interior location of barrier connected to a non-deformable slab.

**Figure 7:**
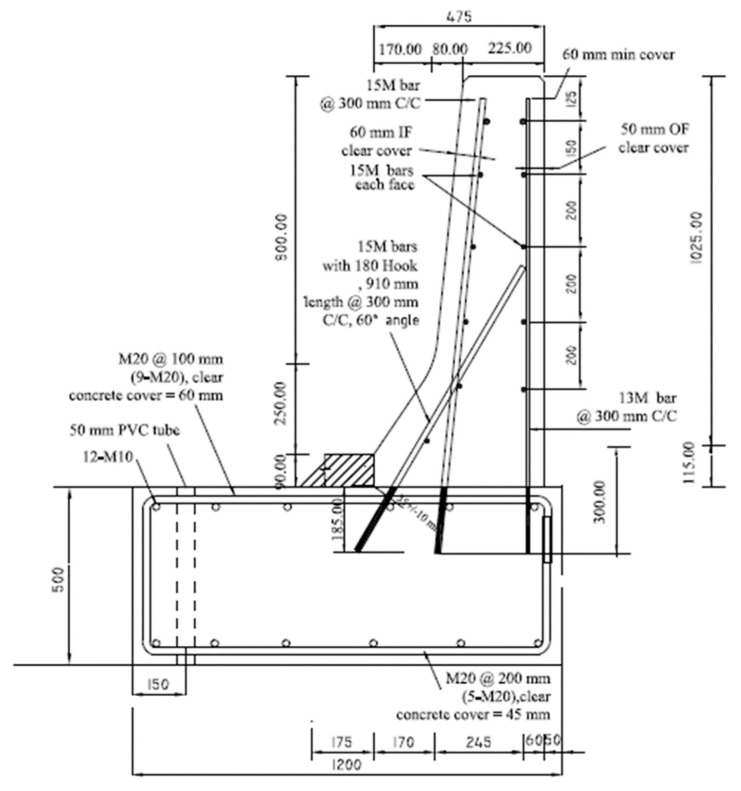
Specimen # 5: Interior location of the barrier connected to a non-deformable slab using post-installed bars.

**Figure 8:**
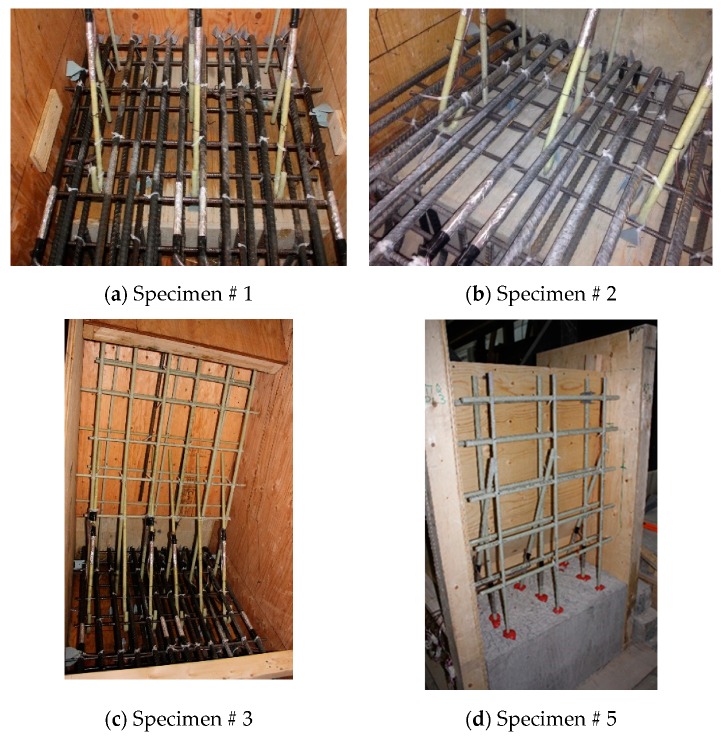
Views of the formwork and reinforcement details. (**a**) Specimen # 1; (**b**) Specimen # 2; (**c**) Specimen # 3; (**d**) Specimen # 5.

Five TL-5 barrier specimens of 900 mm length were constructed and tested by the authors to collapse at Ryerson University’s Structures laboratory to determine their failure patterns and load carrying capacities. Figure 3, Figure 4, Figure 5, Figure 6 and Figure 7 show cross-section dimensions and GFRP bar arrangement for the five specimens (Specimens # 1 through # 5). The difference between Specimen # 1 and # 2 was the orientation of the 180° hooks embedded in the deck slab, which were towards the roadway and towards the outer face of the barrier in Specimens # 1 and # 2, respectively (both specimens represent the interior location in parapets). In these specimens, the vertical GFRP bars at the front and back faces of the barrier wall were made of 15M and 13M bars at 300 mm spacing, respectively, representing the interior segment of a barrier wall. Specimen # 3 showed the end segment of a barrier, with double vertical reinforcement at the front face only, while bar size and spacing in the barrier wall were otherwise identical to those in Specimens # 1 and 2. It should be noted that Specimens # 1 through 3 rested over a deck slab cantilever of length 700 mm, representing the case of slab-on-girder bridge barrier construction. On the other hand, a barrier wall may be built over a non-deformable slab, which is the case of barrier walls cast over voided slab, solid slab, or side-by-side box beam bridges. Specimen # 4 was similar to Specimen # 1, except that the barrier wall was built integrally over a 500 mm thick slab. Specimen # 5 was built to showcase the implementation of replacing the barrier wall in existing bridges with a post-installed barrier wall, using post-installed GFRP bars. HIT RE 500 epoxy was used for post-installation in this case. Figure 8 shows views of the formwork and bar arrangement for each barrier specimen.

**Figure 9:**
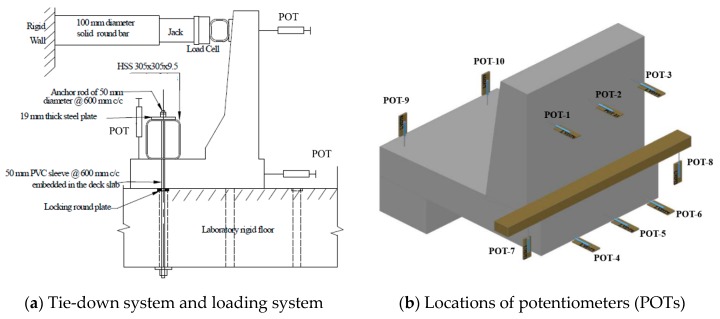
Test setup. (**a**) Tie-down system and loading system; (**b**) locations of potentiometers (POTs).

Based on tested concrete cylinders on the day of casting, the average compressive strengths of concrete were 43, 40, 39, 49 and 52 MPa for Specimens # 1 to # 5, respectively, while the characteristic concrete strengths were 37, 35, 38, 45 and 44 MPa, respectively [4]. Each barrier specimen was tied down to the strong floor in the structures lab according to the test setup shown in Figure 9a. Strain gauges were installed in a few GFRP bars and on the concrete surface to record strains at critical locations with increase in applied transverse loading. Figure 9b shows the arrangement of potentiometers (POTs) to record lateral deflection of the barrier wall, vertical deflection of the cantilever slab, specimen uplift at the tie-down location, and transverse movement of the deck slab. The transverse loading was applied in increments at 990 mm from the top surface of the slab until collapse. The specimen was considered failed when it could not take any more load.

## 3. Experimental Results and Discussions

In Table 2, the maximum transverse loads and resisting moments resulting from the tests are presented and compared to the factored design moments according to CHBDC [17]. The resisting moments were calculated based on the 900 mm width of the barrier and the distance of the applied load from the top surface of the slab, which was 990 mm. The capacity-to-demand ratios (CDR), or the factor of safety in design based on experimental findings, were calculated by dividing the experimental resisting moments by the factored design moments in each case. The CDRs were also calculated based on a material resistance factor of 0.75 [17].

**Figure 10:**
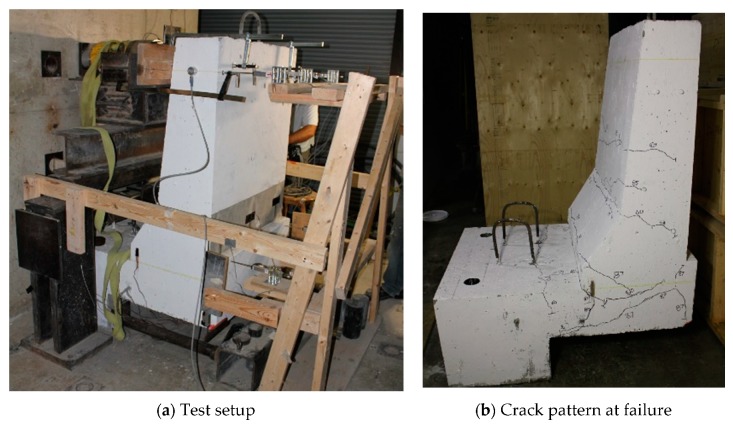
Specimen # 1 before and after testing. (**a**) Test setup; (**b**) crack pattern at failure.

**Figure 11:**
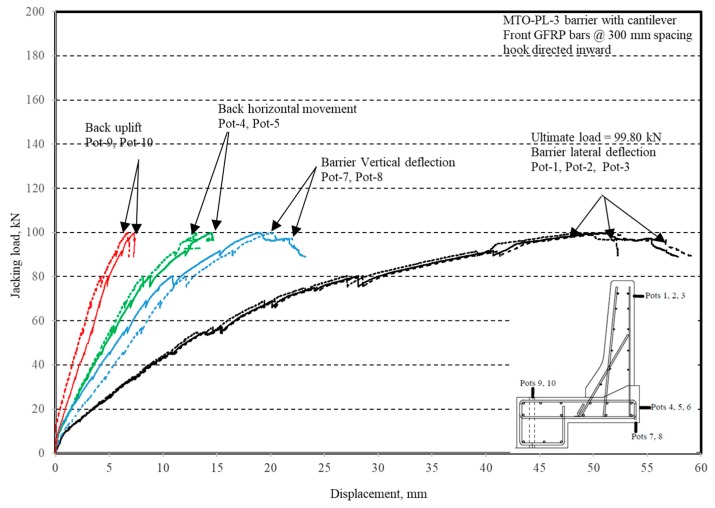
Specimen # 1: Load vs. displacement history.

**Figure 12:**
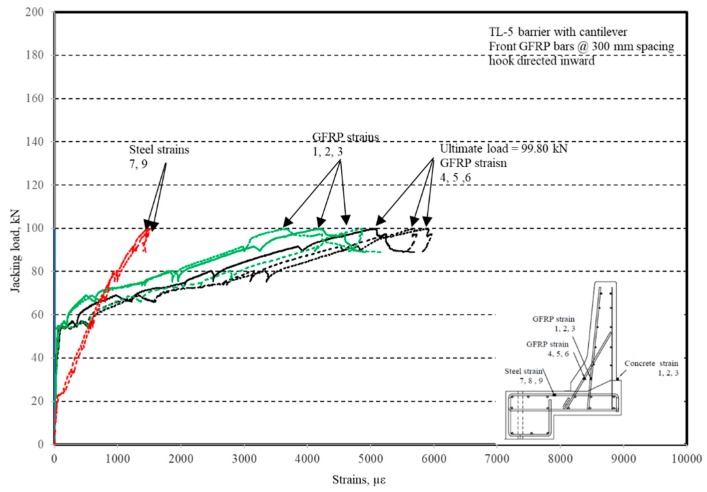
Specimen # 1: Load vs. bar strain history.

**Figure 13:**
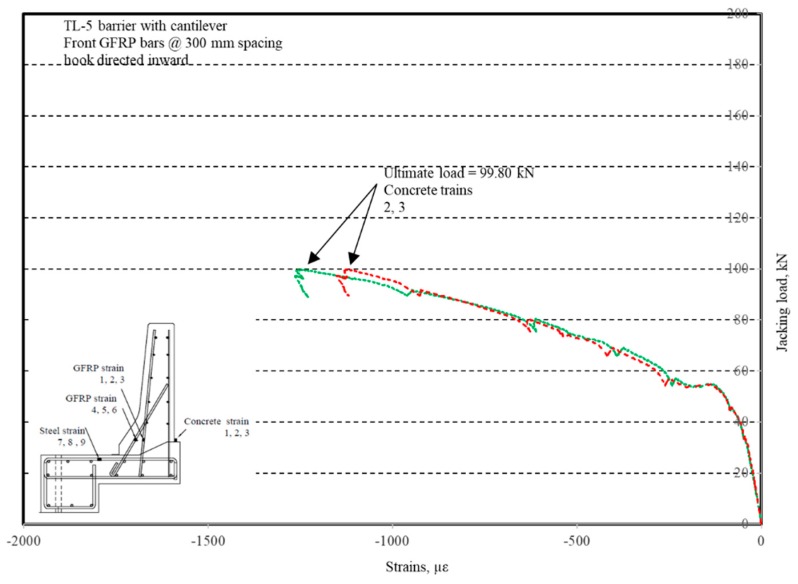
Specimen # 1: Load vs. concrete strain history.

**Figure 14:**
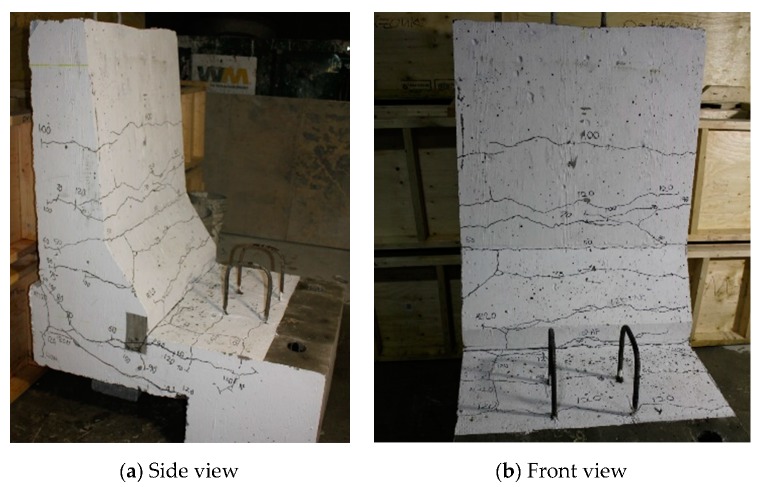
Specimen # 2 crack pattern. (**a**) Side view; (**b**) front view.

The photos of crack patterns at failure and test setups for Specimens # 1 to # 5 are shown in Figure 10, Figure 14, Figure 18, Figure 22 and Figure 26, respectively. Table 3 summarizes the concrete cracking history during the tests. The load–displacement relationship for Specimen # 1 (shown in Figure 10) is depicted in Figure 11. It can be observed that the average deck slab uplift and the horizontal movement at failure were equal to about 7.0 and 13.6 mm, respectively, which was acceptable as they were not affecting the structural behavior of the barrier. The maximum lateral and vertical deflections of the barrier wall at failure were recorded as 50.78 and 19.59 mm, respectively. Tensile strains in the diagonal GFRP bars at the front face are shown in Figure 12, where strain gauges were located at 115 mm above the top surface of the cantilever slab. Average strain in the hooked bars and the adjacent middle bars at failure were recorded as 5487 and 4325 µε, compared to ultimate strain of the GFRP bars of 20,000 µε (per the manufacturer’s document). The relationship between load and strain in the concrete is shown in Figure 13, as obtained from strain gauges located 115 mm above the top surface of the cantilever slab and at the front face of the barrier wall. Concrete compressive strain at failure was 1193 µε, compared to ultimate concrete strain at failure of 3500 µε. The tensile strain at failure in the steel bars at the fixed end of the cantilever deck slab was 1461 µε, compared to the steel yield strain of 2000 µε. The failure of the barrier–deck junction was due to diagonal tension cracking in the deck slab cantilever just under the barrier wall, as depicted in Figure 10b.

**Figure 15:**
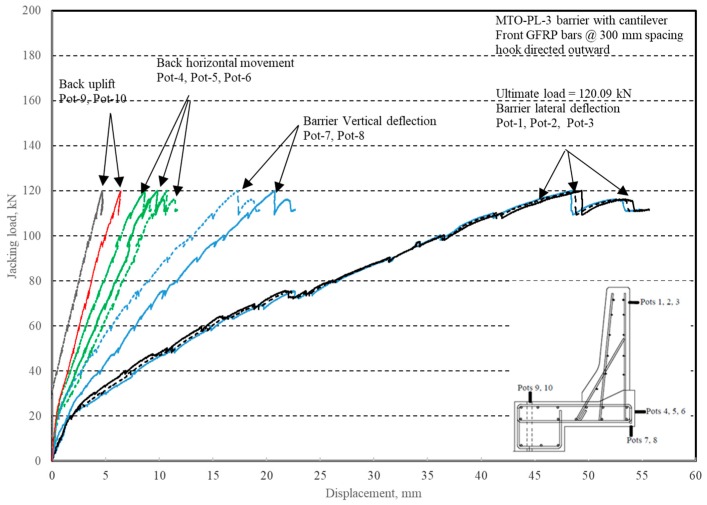
Specimen # 2: Load vs. displacement history.

**Figure 16:**
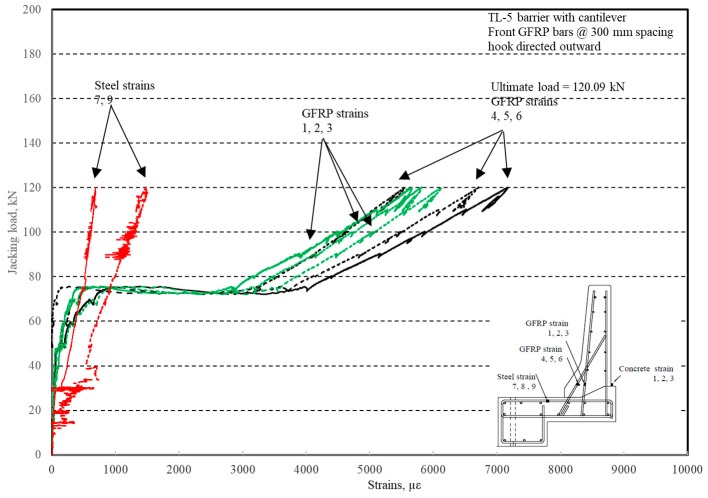
Specimen # 2: Load vs. bar strain history.

**Figure 17:**
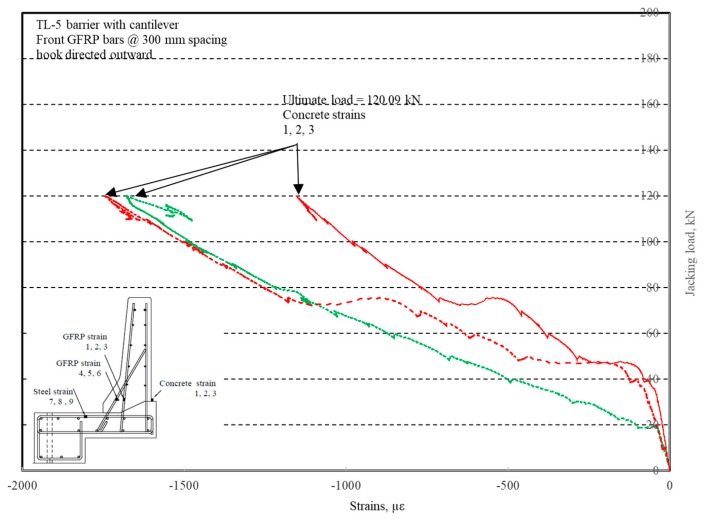
Specimen # 2: Load vs. concrete strain history.

The load–displacement relationship for Specimen # 2 (shown in Figure 14) is depicted in Figure 15. One may observe that the average deck slab uplift and the horizontal movement at failure were equal to about 5.54 and 9.72 mm, respectively, which was acceptable as they were not affecting the structural behavior of the barrier. Maximum lateral deflection of the barrier wall at failure was 48.80 mm and the barrier vertical deflection was 19.02 mm. Tensile strains in the diagonal GFRP bars at the front face are shown in Figure 16. Average strains in the hooked bars and the adjacent middle bars at failure were 6564.6 and 5780.3 µε, compared to ultimate strain of the GFRP bars of 20,000 µε. The relationship between load and strain in the concrete is shown in Figure 17. Concrete compressive strain at failure was recorded as 1524 µε, compared to ultimate concrete strain at failure of 3500 µε. The tensile strain at failure in the steel bars at the fixed end of the cantilever deck slab was 1001.5 µε, compared to the steel yield strain of 2000 µε. Failure of the barrier–deck junction was due to diagonal tension in the deck slab cantilever just under the barrier wall. One may observe that the barrier–deck anchorage capacity increased by 20% only when the hooks faced the outer face of the barrier, compared to the arrangement on which the hooks faced towards the traffic side in Specimen # 1. This may be attributed to greater confinement to the concrete in the deck slab under the barrier wall associated with orienting the hook to the back side of the wall, thus delaying the diagonal tension failure in this region to a higher load.

**Figure 18:**
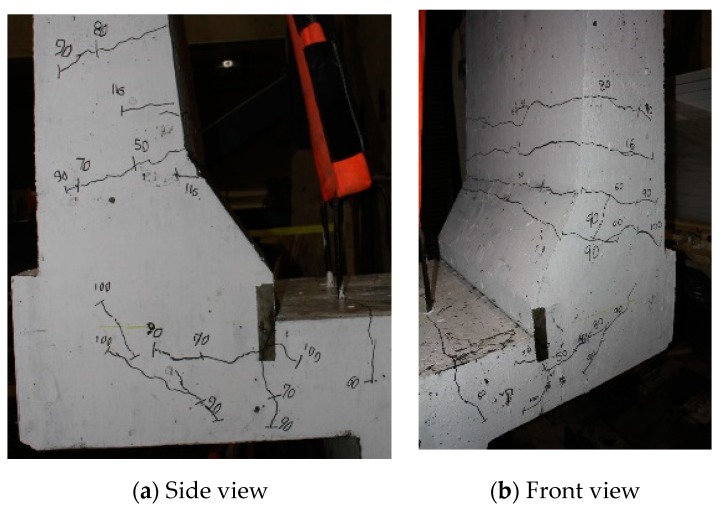
Specimen # 3: crack pattern. (**a**) Side view; (**b**) front view.

**Figure 19:**
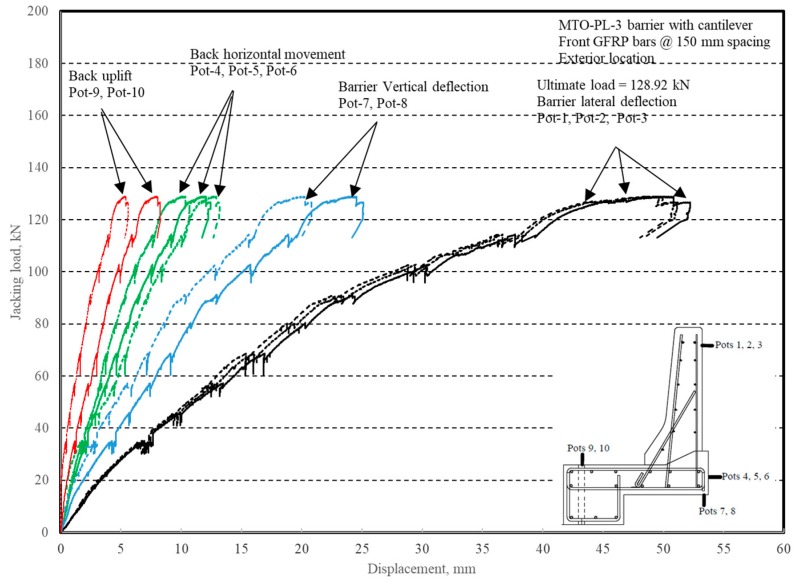
Specimen # 3: Load vs. displacement history.

**Figure 20:**
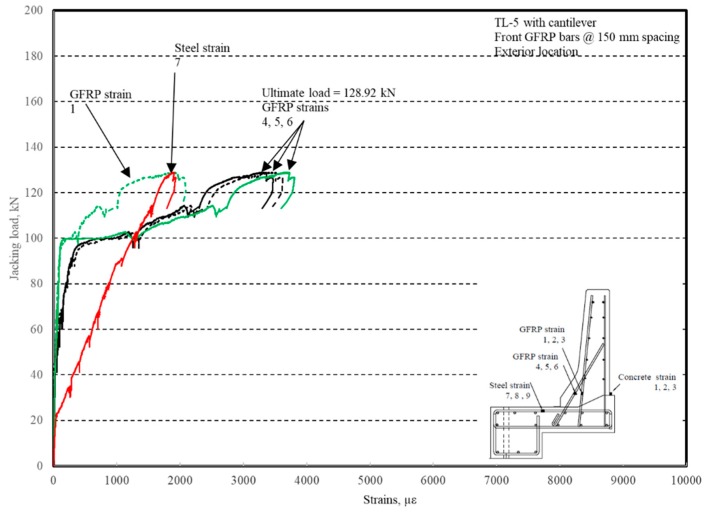
Specimen # 3: Load vs. bar strain history.

**Figure 21:**
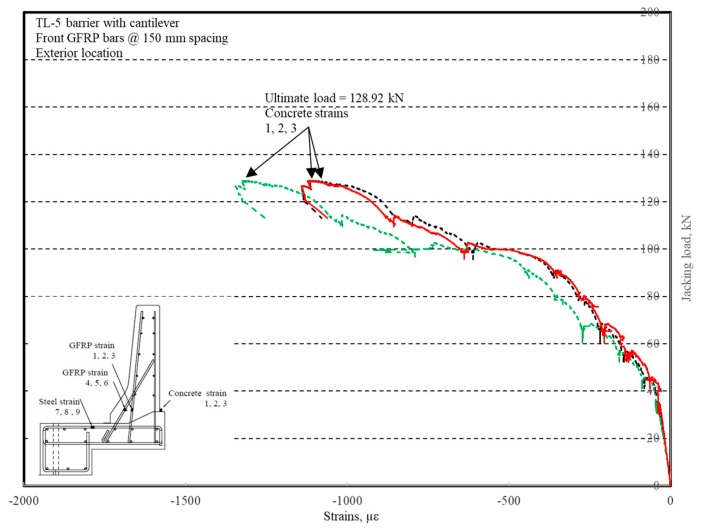
Specimen # 3: Load vs. concrete strain history.

The load–displacement relationship for Specimen # 3 (shown in Figure 18) is depicted in Figure 19. It was noted that the average deck slab uplift and the horizontal movement at failure were equal to 6.56 and 11.51 mm, respectively, which was acceptable as they were not affecting the structural behavior of the barrier. Maximum lateral deflection of the barrier wall at failure was 49.06 mm and the barrier vertical deflection was 21.97 mm. Tensile strains in the diagonal GFRP bars at the front face are shown in Figure 20. It was observed that the average strains in the hooked bars and the adjacent middle bars at failure were 3459 and 1874 µε, compared to ultimate strain of the GFRP bars of 20,000 µε. The relationship between load and strain in the concrete is shown in Figure 21. Concrete compressive strain at failure was recorded as 1238 µε, compared to ultimate concrete strain at failure of 3500 µε. Failure of the barrier–deck junction was due to diagonal tension cracking in the deck slab cantilever just under the barrier wall. The tensile strain at failure in the steel bars at the fixed end of the cantilever deck slab was 1839 µε, which gives an indication that the steel bars behaved in the elastic range at specimen failure.

**Figure 22:**
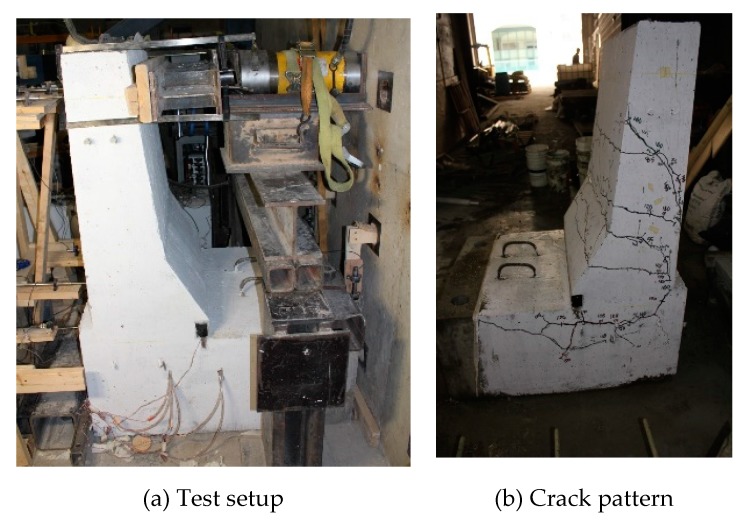
Specimen # 4 before and after testing. (**a**) Test setup; (**b**) crack pattern.

**Figure 23:**
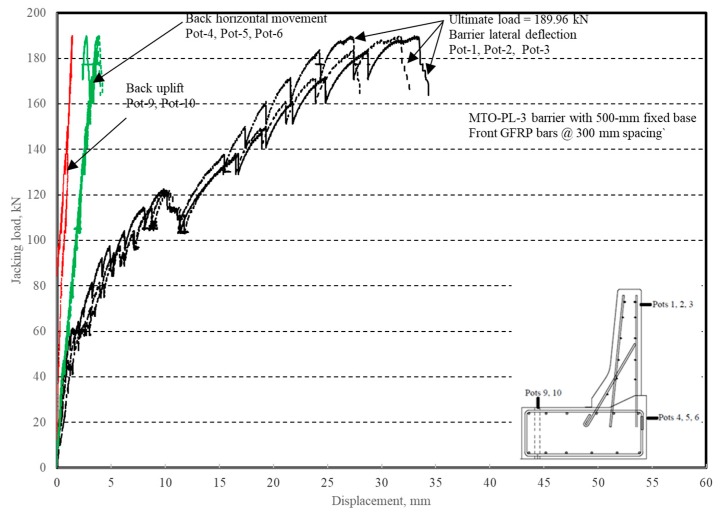
Specimen # 4: Load vs. displacement history.

**Figure 24:**
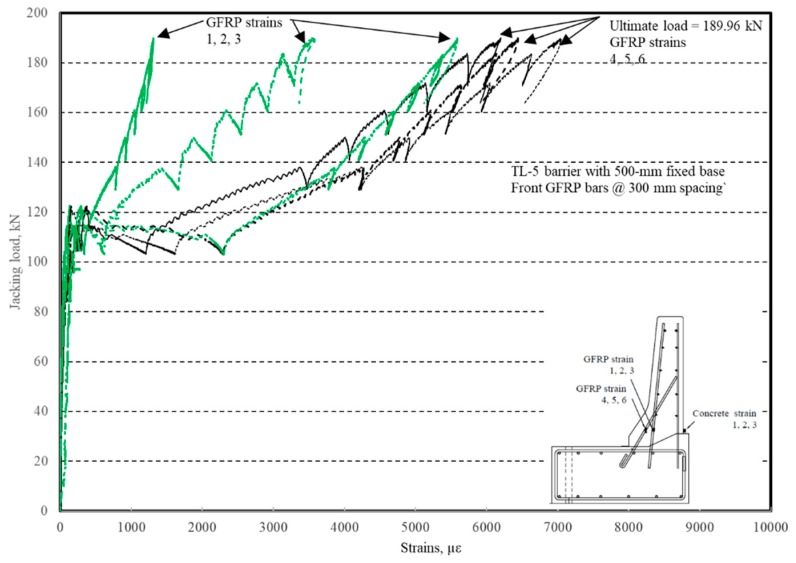
Specimen # 4: Load vs. GFRP bar strain history.

**Figure 25:**
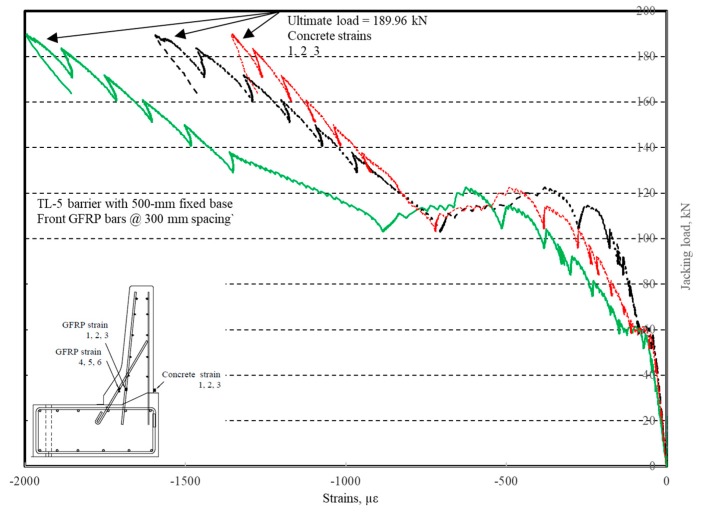
Specimen # 4: Load vs. concrete strain history.

The load–displacement relationship for Specimen # 4 (shown in Figure 22), with a 500 mm thick deck slab, is depicted in Figure 23. The average deck slab uplift and the horizontal movement at failure were recorded as 1.40 and 3.42 mm, respectively, which was acceptable as they were not affecting the structural behavior of the barrier. Maximum lateral deflection of the barrier wall at failure was 30.50 mm, which was very much smaller than that for Specimen # 1 with a cantilever deck slab. Tensile strains in the diagonal GFRP bars at the front face are shown in Figure 24. Average strains in the hooked bars and the adjacent middle bars at failure were 6558 and 3477 µε, compared to ultimate strain of the GFRP bars of 20,000 µε. The relationship between load and strain in the concrete is shown in Figure 25. Concrete compressive strain at failure was 1647 µε, compared to ultimate concrete strain at failure of 3500 µε. Failure of the barrier–deck junction was due to sudden concrete breakout at the location of the embedded GFRP bars in the deck, as depicted in Figure 22b.

**Figure 26:**
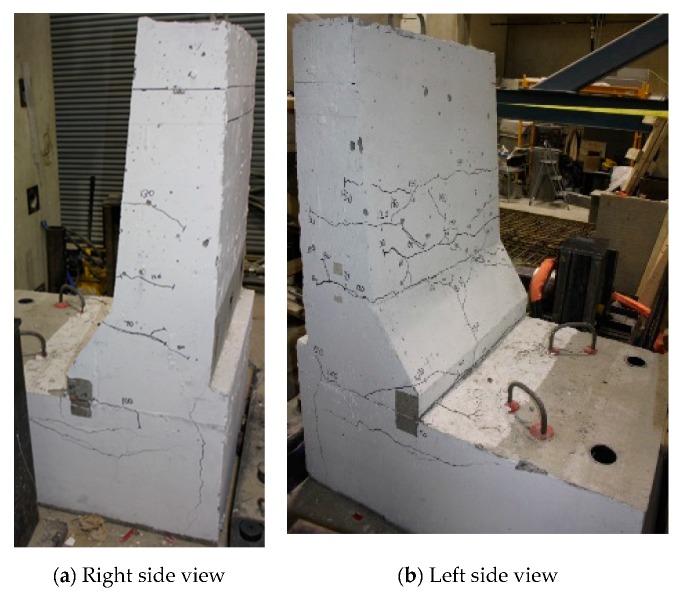
Specimen # 5: crack pattern. (**a**) Right side view; (**b**) left side view.

**Figure 27:**
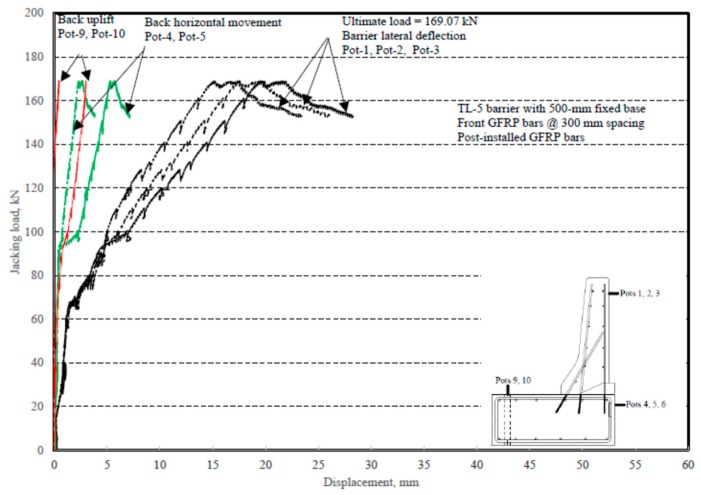
Specimen # 5: Load vs. displacement history.

**Figure 28:**
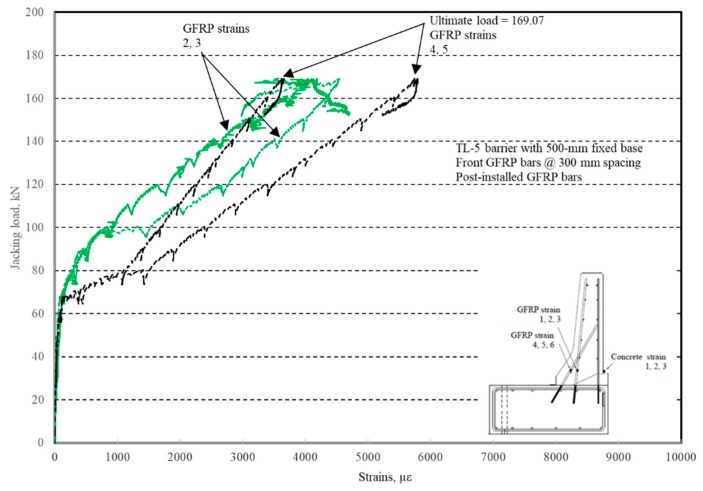
Specimen # 5: Load vs. GFRP bar strain history.

**Figure 29:**
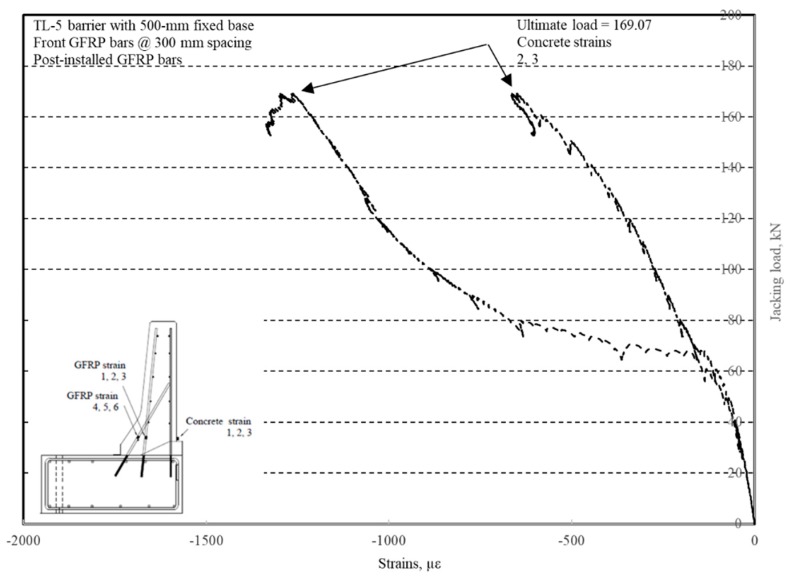
Specimen # 5: Load vs. concrete strain history.

The load–displacement relationship for Specimen # 5 (shown in Figure 26) is depicted in Figure 27. It was observed that the average deck slab uplift and the horizontal movement at failure were equal to 1.71 and 4.18 mm, respectively. Maximum lateral deflection of the barrier wall at failure was 19.38 mm. Tensile strains in the diagonal GFRP bars at the front face are shown in Figure 28. Average strains in the hooked bars and the adjacent middle bars at failure were 4387 and 4113 µε, compared to ultimate strain of the GFRP bars of 20,000 µε. The relationship between load and strain in the concrete is showcased in Figure 29. Concrete compressive strain at failure was 981 µε, compared to ultimate concrete strain at failure of 3500 µε. Failure of the barrier–deck junction was sudden concrete breakout at the location of the embedded GFRP bars in the deck, as depicted in Figure 26.

Azimi et al. [18] conducted experimental tests to collapse on short barriers to examine their load carrying capacities and failure modes at the barrier–deck anchorage when reinforced with ribbed-surface GFRP bars with headed ends produced in Europe. Most recently, Rostami et. al. [19] conducted similar research to that presented in this paper, but using sand-coated GFRP bars with headed ends produced by the same manufacturer supplying the bars for the current research. Rostami et al. (2017b) also conducted testing identical to that presented in this paper using GFRP bars with a spiral-profiled surface and a 180° hook of 115 mm inside diameter, produced by another Canadian manufacturer. Moreover, Dervishhasani and Sennah [20] conducted similar research using GFRP bars with headed ends, developed by a third Canadian manufacturer. The four types of GFRP bars mentioned above are produced with different surface profiles, anchorage details, tensile strength, modulus of elasticity, and stain at rupture. However, all of them meet the requirement for being identified as Grade III GFRP bars, with a minimum tensile strength of 1000 MPa and a modulus of elasticity of 60 GPa [21]. In all these tests, the experimental barrier capacities were greater than or equal to the design values specified in the Canadian Highway Bridge Design Code [4] after applying a proper resistance factor for materials. The failure modes observed in these tests included (i) diagonal tension crack in the deck slab region under the barrier wall in the case of deck slab cantilevers, (ii) anchorage cracking of the slab under the barrier wall in the case of a non-deformable barrier base, and (iii) diagonal tension cracking (shear failure) in the barrier wall between the transverse load location and the interfaces between the two tapered portions of the barrier wall. It should be noted that there is no available design procedure for such failure mode in North American bridge design codes. As such, experimental testing is the only available methodology to qualify the use of GFRP bars with special anchorage profile at the barrier–deck anchorage zone.

## 4. Comparison with Finite Element (FEA) Results

**Figure 30:**
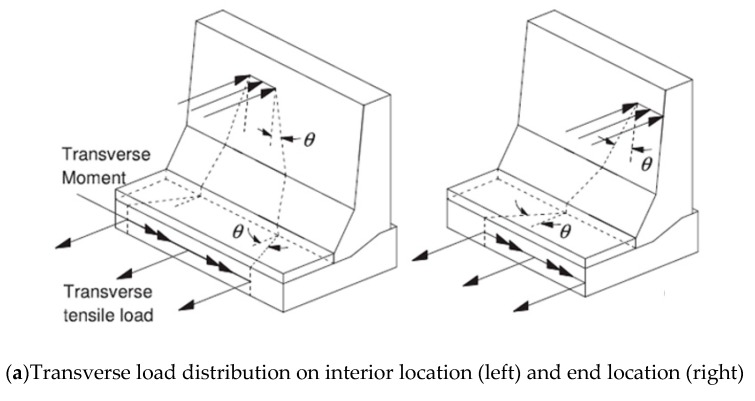

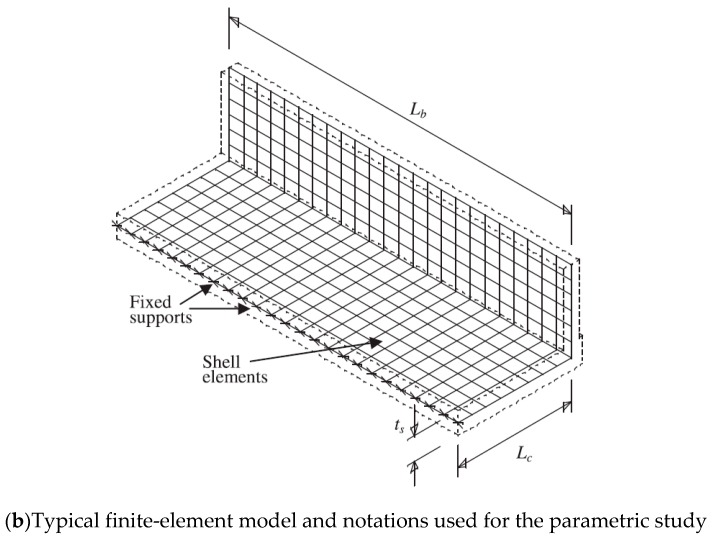
Finite element model and applied transverse loading. (**a**)Transverse load distribution on interior location (**left**) and end location (**right**). (**b**) Typical finite element model and notations used for the parametric study [18].

When an errant vehicle collides with a bridge barrier, the lateral impact force is distributed in the barrier wall and the deck slab with dispersal angles, leading to the design forces at the barrier–deck junction specified in CHBDC commentaries [18]. Figure 30a shows the distribution of a vehicle transverse impact load on the concrete barriers [18]. Design shear forces and bending moment at the barrier–deck interface can be calculated by finite element modeling for a 1000 mm barrier length. The length of cantilever slabs of bridges can also be different, as in slab-on-girder or non-deformable bases. Therefore, the change in the support condition for barriers should be studied. Barrier length is the distance between two free ends of the barrier or between expansion joints. The length of the barrier affects the dispersion angle of applied forces (Figure 30a). Finite element analysis (FEA) was conducted by Azimi et al. [17] to examine the effects of barrier length, deck slab thickness, and cantilever length on the factored applied moment at the barrier–deck junction of TL-5 barriers, as affected by the barrier length, deck slab cantilever length, and deck slab thickness.

Slab thickness, t_s_, cantilever overhang length, L_c_, and the barrier length, L_b_, were considered in the parametric study by Azimi et al. [17]. The associated values for each parameter were: 175, 225, 275, and 350 mm for t_s_; 0, 0.5, 1.0, 1.5, and 2.0 m for L_c_; and 3, 4, 6, 8, 10, and 12 m for L_b_. Linear elastic FEA was performed on the TL-5 barrier of Figure 1. Shell element with six degrees of freedom at each node was used with a change in thickness to model TL-5 barriers with tapered faces. The maximum element sizes were taken as 50 × 50 mm, with an aspect ratio of 1.2 in a few cases. A view of the FEA model is shown in Figure 30b. The support condition was taken as fixed at the end of the cantilever overhang. The cantilever length, L_c_, of 0 represented a fixed base of the barrier wall, for a non-deformable deck slab. Barrier lengths were considered between 3 and 12 m, as this study showed that greater length does not have considerable effect on the results. The reason to choose these length is that the minimum barrier length, L_b_, is usually considered to be 3 m in practice, and the maximum barrier length of 12 m was taken into consideration in this study because analysis showed that greater lengths would have insignificant effect on the distribution of forces in the inner portions of the barriers, and no effect at the end portions of the barrier. Transverse loads were applied at the middle of the barrier for interior locations and at the end of barrier for the exterior locations, and were distributed over specified lengths. An equation for factored design moments at the barrier–deck interface was developed based on the statistical package of curve fitting (Table 4), to determine the factored design moment at the barrier–deck junction as a function of barrier length and deck slab cantilever length and thickness, which can be used only for the range of parameters considered here.

**Figure 31:**
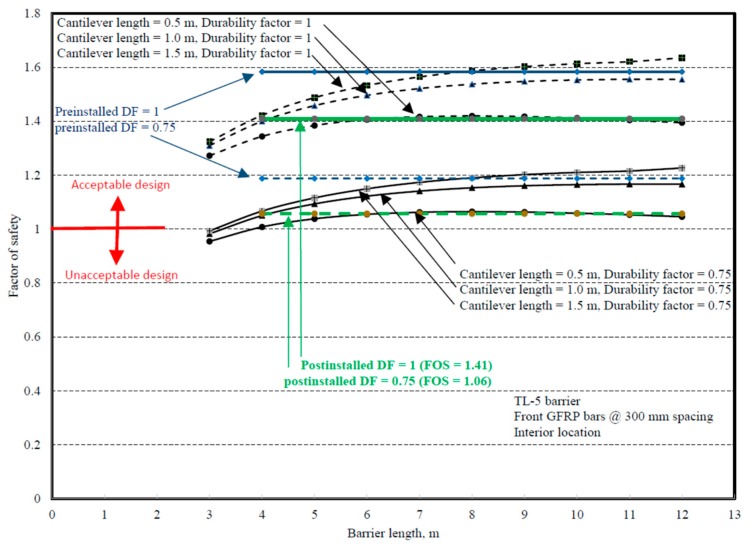
Design factors of safety for TL-5 barrier in Figure 1b at an interior location.

**Figure 32:**
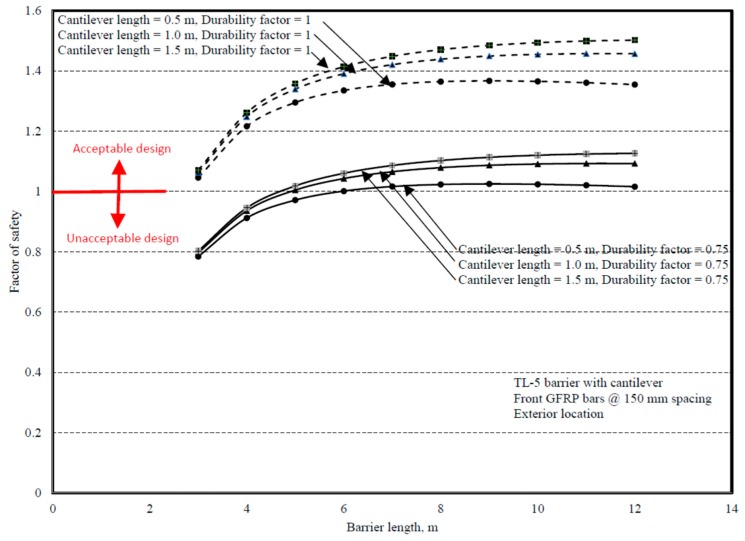
Design factors of safety for TL-5 barrier in Figure 1b at end location.

Comparisons between experimental and numerical results for the interior and end locations are shown in Figure 31 and Figure 32. Chapter 2 of the CHBDC [4] specifies that the designer shall consider the environmental conditions and deterioration mechanisms for FRP reinforcement, and recommends that a 0.75 durability factor be applied to the results, which was followed by authors in this paper. To be able to qualify the proposed GFRP bar detailing in Figure 1b, the factor of safety was considered at least 1 to ensure that the experimental capacity was at least equal to the factored applied moment at the barrier deck junction specified in CHBDC or obtained by FEA modeling. Figure 31 and Figure 32 present the factors of safety in design of the proposed barrier when a material durability factor of 0.75 was applied.

## 5. Conclusions

Based on the experimental findings in this research, the following conclusions can be made:

- It was observed that using the proposed reinforcement arrangement presented in Figure 1b, utilizing GFRP reinforcement, is safe and practical for barrier lengths greater than or equal to 6 m in the case of slab-on-girder and box girder bridges with deck slab cantilever lengths up to 2 m.
- Regarding embedment length for the vertical bars, it was observed that 185 mm vertical embedment length for the GFRP bars in the deck slab was sufficient and satisfied the required barrier–deck anchorage capacity. The same GFRP bar arrangement can be used in solid slab and voided-slab bridge cross-sections with a minimum deck slab thickness of 500 mm.
- The barrier–deck anchorage capacity increased by 20% only when the 180° hooks were oriented towards the outer face of the barrier, compared to the arrangement in which these hooks faced toward the traffic side of the barrier wall.
- Tensile strains in the diagonal GFRP bars at the front face in the hooked bars and the adjacent middle bars at failure were far below the ultimate strain of the GFRP bars. Additionally, the concrete compressive strain at failure in the outer face of the barrier was also far below the ultimate concrete strain at failure. Moreover, the tensile strain at failure in the steel bars at the fixed end of the cantilever deck slab was below the steel yield strain. These observations support the observed failure mode at the barrier–deck region.
- Failure of the barrier specimen with deck slab cantilever was due to diagonal tension cracking in the deck slab cantilever just under the barrier wall. On the other hand, failure in the barrier specimen supported over the 500 mm thick deck slab was due to sudden concrete breakout at the location of the embedded GFRP bars in the deck.

## Figures and Tables

**Table 1:** Properties of GFRP bars as obtained from the manufacturer.

Product Name	Bar Size	Tensile Strength (MPa)	Elastic Modulus, E (GPa)	Failure Strain	Nominal Cross Section Area (mm^2^)	Gross Cross Section Area (mm^2^)
Straight bar	# 4 (M13)	1281.5	61.32	2.09%	129.0	145.0
	# 5 (M15)	1237.4	60.01	2.06%	199.0	224.4
Straight portion of the hooked bar	# 5 (M15)	1500.0	60.10	2.45%	199.0	233.0

**Table 2:** Experimental results.

Design Values	Specimen # 1	Specimen # 2	Specimen # 3	Specimen # 4	Specimen # 5
Load at failure, kN (experiment)	99.80	120.09	128.92	189.96	169.07
Resisting moment, kN.m/m (experiment)	109.78	132.10	141.81	208.96	185.98
Factored design moment, kN.m/m (CHBDC, 2006b)	83.00	83.00	102.00	83.00	83.00
Capacity-to-demand ratio: CDR (2)/(3)	1.32	1.59	1.39	2.52	2.24
CDR Ratio (2)/(3) with 0.75 resistance factor	0.99	1.19	1.04	1.89	1.68

**Table 3:** Crack pattern history.

Load and Crack Pattern	Specimen # 1	Specimen # 2	Specimen # 3	Specimen # 4	Specimen # 5
Load at first visible crack, kN	50	40	50	50	60
Location of first visible crack	At intersection of tapered portions of front side of barrier wall	At barrier–deck junction	At barrier–deck junction and intersection of tapered portions of front side of barrier wall	At intersection of tapered portions of front side of barrier wall	At barrier–deck junction
Load at next step flexural cracks, kN	60	70	60	90	70
Location of next step flexural cracks	At barrier–deck junction and deck slab	At deck slab	At deck slab	At barrier–deck junction	At intersection of tapered portions of front side of barrier wall
Load at failure, kN	99.80	120.09	128.92	189.96	169.07
Crack pattern at failure	Propagation of initial cracks in both barrier and slab, plus sudden diagonal tension crack in deck	Propagation of initial cracks in both barrier and slab, plus sudden diagonal tension crack in deck	Propagation of initial cracks in both barrier and slab, plus sudden diagonal tension crack in deck	Propagation of initial flexural cracks and sudden concrete breakout at the location of embedded GFRP bars in deck	Propagation of initial flexural cracks and sudden concrete breakout at the location of embedded GFRP bars in deck

**Table 4:** Factored design moments at the barrier–deck interface [18].

Design Parameters		Design Values
Lateral Load, P_t_ (kN)		210
Factored lateral load (kN)		357
Length of lateral load (mm)		2400
Height of lateral load, H (mm)		990
$M_{inner}$ (kN.m)	Fixed base	132
	Cantilever deck slab	$\begin{array}{l} 100{\left(L_{b}+2.3t_{s}\right)}^{-1}\\ +2.83t_{s}^{0.2}{\left(L_{b}-1\right)}^{0.7}L_{c}^{-0.8}\\ +143t_{s}+23 \end{array}$
$M_{end}$ (kN.m)	Fixed base	148
	Cantilever deck slab	$\begin{array}{l} 14t_{s}^{-1}{\left(L_{b}+2.3t_{s}-2\right)}^{-1}\\ +2.83t_{s}^{0.2}{\left(L_{b}-1\right)}^{0.7}L_{c}^{-0.7}\\ +240t_{s}+25 \end{array}$

Notes: Formulas are best applicable for: $175\;mm\leq t_{s}\leq 350\;mm$; $0\leq L_{c}\leq 2.0\;m$; $3.0\;m\leq L_{b}$ (*t_s_* = overhang thickness (m); *L_c_* = cantilever length (m); *L_b_* = barrier length (m); *M_inner_* = moment in the interior locations; *M_end_* = moment in the end locations).
